# Relationship between alexithymia, loneliness, resilience and non-suicidal self-injury in adolescents with depression: a multi-center study

**DOI:** 10.1186/s12888-023-04938-y

**Published:** 2023-06-19

**Authors:** Bing Zhang, Wei Zhang, Lingmin Sun, Cheng Jiang, Yongjie Zhou, Kongliang He

**Affiliations:** 1 School of Mental Health and Psychological Sciences, Anhui Medical University, Hefei, 230022 China; 2 Affiliated Psychological Hospital of Anhui Medical University, Hefei, 230022 China; 3 Anhui Mental Health Center, 230022 Hefei, China; 4 Hefei Fourth People’s Hospital, Hefei, China; 5 Shenzhen Kangning Hospital, Shenzhen, China; 6 Shenzhen Mental Health Center, Shenzhen, Guangdong China; 7 Psychological counseling department, Hefei Fourth People’s Hospital, Anhui, 230000 China

**Keywords:** Adolescents, Alexithymia, Loneliness, Non-suicidal self-injury, Resilience

## Abstract

**Objective:**

Non-suicidal self-injury (NSSI) behaviors are prevalent in adolescents and have adverse effects on physical and mental health. However, little is known about the relationship between NSSI and alexithymia, or the underlying mechanisms that could explain this relationship. This study aimed to elucidate the current status of NSSI in adolescent depression, and analyze the relationship between alexithymia, loneliness, resilience, and adolescent depression with NSSI, so as to provide a theoretical basis for psychotherapeutic interventions.

**Method:**

The study sample involved inpatients and outpatients from 12 hospitals across China and adolescents with depression who met the DSM-5 diagnostic criteria for depression episode. The following scales were used: The Functional Assessment of Self-Mutilation, Toronto Alexithymia Scale, UCLA Loneliness Scale, and Connor Davidson Resilience Scale.

**Results:**

The detection rate of NSSI in adolescents with depression from 2021.01.01-2022.01.01 was 76.06% (1782/2343). Spearman’s correlation analysis revealed a significant correlation between alexithymia, loneliness, resilience and NSSI in depressed adolescents, and the results of the non-parametric test showed that the differences between the two groups for each factor were statistically significant. Binary logistic regression results showed that alexithymia (B = 0.023, p = 0.003, OR = 1.023, 95% CI: 1.008–1.038) and depression (B = 0.045, p < 0.001, OR = 1.046, 95% CI: 1.026–1.066) are risk factors for NSSI, resilience (B = − 0.052, p < 0.001, OR = 0.949, 95% CI: 0.935 − 0.964) is a protective factor for NSSI. Alexithymia directly predicted NSSI and also indirectly influenced NSSI through the mediated effect of resilience. Loneliness moderates the first half of the path of this mediated model.

**Conclusion:**

The present study confirms a moderated mediation effect: Alexithymia can have an impact on NSSI behaviors in depressed adolescents through the mediating role of resilience. Loneliness, as a moderating variable, moderated the first half of the pathway of the mediating model. We discuss perspectives for future research and interventions based on the findings of the study.

## Introduction

Non-suicidal self-injury (NSSI) is the act of an individual deliberately and repeatedly injuring his or her own bodily tissues without clear suicidal intent, commonly in the form of cuts, bruises, burns, scratches, bites, hitting the head against a hard object, etc. These acts are not fatal but are extremely dangerous [1]. NSSI has a high global prevalence, especially occurring in adolescents, for example, the detection rate of NSSI behaviors among adolescents in the US community ranged from 13.0 to 46.5%, whereas the detection rate among adolescents in Canada was 17.0%, 6.2% in Australia [2], and 27.4% in China [3]. The recurrence rate of NSSI is high and increasing year by year [4], which has seriously endangered the physical and mental health of adolescents [5]. NSSI has been shown to be strongly associated with suicidal behavior [6, 7], increasing the risk of suicidal behavior sevenfold [8] and being a significant predictor of suicidal behavior in adolescents [2], which has become a global concern for public health concern [9].

Clinical treatment options for depressed adolescents with NSSI behaviors include medication (e.g. SSRIs), psychotherapy (e.g. cognitive behavioral therapy (CBT), Discriminative Behavioral Therapy (DBT)) and physiotherapy (e.g., repetitive transcranial magnetic stimulation (r-TMS)), but these options are not clinically effective [10]. Parents are reluctant to give their children medication due to concerns about side-effects, adolescents find it difficult to attend psychotherapeutic sessions due to academic pressure, and parents’ lack of knowledge about physiotherapy may also make them more resistant to seeking this treatment option. This is why it is important to find a simple, effective and targeted intervention.

### Alexithymia in NSSI

Alexithymia, a term initially coined by Nemiah and Sifneos [11], is not an independent disorder, but a psychological trait that accompanies the development of personality [12]. Taylor et al. explored the development of alexithymia based on psychoanalytic theory, and they suggested that the degree of an individual’s alexithymia is relatively stable in clinical presentation as well as in other interpersonal contexts, while it is the ability to partially psychologies that may change, particularly in the context of empathy or other emotionally stressful relationships [13]. Barberis et al. explored the development of alexithymia based on attachment theory. They identified alexithymia as a major risk factor for mental health, and their findings showed that higher attachment anxiety and attachment avoidance predicted certainty and uncertainty about mental states, leading to higher alexithymia, and that higher levels of alexithymia were also associated with higher levels of psychopathology and dysfunctional interpersonal representations [14]. Barberis et al. explored the development of alexithymia based on self-determination theory, where they noted that parental control over the inner workings of the adolescent can hinder the development of a secure and positive sense of self, leading to the frustration of their basic psychological needs, which is one of the core processes that often occurs in adolescents’ physical and mental development, resulting in higher levels of alexithymia [15].

Alexithymia can also be a transient state and the severity of alexithymia can vary at different levels of stress, anxiety and depression [16]. The increased risk of suicide in depressed patients is strongly correlated with alexithymia, according to numerous studies [17]. According to international research, alexithymia is one of the potential risks that is most closely related to self-harm [18–20]. Because alexithymia people’s cognitive approach is conditioned by external stimuli, they suffer from difficulty comprehending and communicating their emotions, have difficulty thinking extrovertedly, and have difficulty with their imagination [21]. According to research, alexithymia in teenagers is highly associated with self-harm [22–24], and the severity of alexithymia is significantly higher in patients with repeated self-injury (> 5 times/year) than in occasional self-injury patients (< 5 times/year)[25]. According to a study, alexithymia was a strong predictor of NSSI at the age of five months, which raises the possibility that depression and alexithymia are also risk factors for NSS [26].

Currently, experience-avoidance theory maintains that NSSI is a coping mechanism used by those who, when exposed to unpleasant stimuli, display a reduced capacity for emotional expression [27]. It has been demonstrated that alexithymia is a significant component that, when combined with troublesome emotions, may increase a person’s propensity to utilize NSSI as an alternate means of expressing their emotions or as a means of escaping unpleasant emotional experiences if they suffer from depression [21]. NSSI is frequently undetectable and challenging to identify early, particularly in teenagers with alexithymia. Adolescents’ mental health will be negatively impacted by long-term, repetitive NSSI, and their risk of suicide will rise significantly.

### The mediated effect of resilience in alexithymia and NSSI

An individual’s sense of continuity with regard to their survival aims is aided by resilience, which is a strong inner protective force. Through the activation of personal growth initiatives, it represents a lateral attitude that can be described as the capacity to overcome challenges encountered in various spheres of life with a persevering attitude and a sound awareness of oneself and one’s inner coherence [28]. Resilience can be viewed as a dynamic process that is always self-regulating and improving as a person matures. It is a person’s mental ability and internal resource. Teenagers who are resilient will know what to do when faced with challenges [29]. While supporting the production of meaning can aid in helping people make sense of their situation and identity, it can also assist them in finding methods to prevent long-term psychological suffering and maintain a normal developmental trajectory [30]. Alexithymia was discovered to be inversely linked with resilience in a study of depressed people [31]. The negative consequences of alexithymia on suicide ideas and conduct can be tempered by using greater psychological resources for those with high levels of resilience [32]. A diminished capacity to actively communicate with others and notice the emotional changes of patients was linked to lower levels of resilience in a correlation study of alexithymia and resilience among nursing undergraduates [33]. Resilience can also actively remodel a person’s psychology, promote a positive and balanced state of mind, and stop the emergence of suicidal ideation [34].

Numerous studies have shown the positive impact of resilience when mediated by various psychological factors. Resilience has a mediated effect between physical fitness and anxiety in adolescents [35], resilience has a mediated effect between school bullying and anxiety in adolescents [36], and resilience has a mediated effect between childhood abuse and suicidal ideation in adolescents [37], according to several Chinese studies. Resilience has been shown to mediate the relationship between alexithymia and stress, and is effective in reducing alexithymia. In particular, it can alleviate other psychological problems associated with high alexithymia, such as significant functional impairment in emotional awareness, social attachment and interpersonal relationships [38]. Additionally, resilience has been demonstrated to buffer the relationship between loneliness and depressive symptoms in elderly residents of nursing facilities [39]. The study discovered that while resilience acts as a mediator between alexithymia and NSSI, alexithymia can strongly predict resilience and NSSI [40]. This is in line with the findings of this study, which show that alexithymia predicts resilience negatively while positively predicting NSSI and that resilience can alter the link between alexithymia and NSSI.

### The relationship between loneliness and other psychological factors

The time between adolescence and early youth is known as the “psychological weaning period,“ according to American psychologist L.S. Hollingworth. Teenagers experience a lot of anxiety during this time. Even if they feel a subjective need and desire for independence, it takes them a while to quickly get used to independent living. There are numerous issues that they are unable to resolve on their own, and they are hesitant to seek their parents or other people’s assistance out of concern that doing so might jeopardize their independence. Additionally, kids are still developing their need for intimacy and the social connections that go along with it, which makes it challenging for them to escape isolation when it happens. During this time, loneliness is a constant, pervasive state of consciousness [41]. According to affective, cognitive, and motivational dimensions, loneliness is a widespread phenomenon that has been linked to depression as a result of its association with unpleasant emotions [42]. Higher levels of loneliness were linked to a higher chance of NSSI, suggesting a relationship between the two [43]. A recent study showed that adolescents who self-injured throughout the lifespan scored higher on parent-related loneliness than adolescents who did not self-injure for life, and that loneliness was significantly and positively associated with emotional regulation of self-injury [44]. According to the NSSI Interpersonal Functional Model, there are significant connections between interpersonal experiences and NSSI [45]. For instance, loneliness is a bad interpersonal experience that frequently causes NSSI [46].

Chronic loneliness jeopardizes a person’s mental health in the future and a variety of indices of psychosocial functioning [47]. Research on loneliness has shown that it is associated with a range of risk or protective factors strongly associated with NSSI, including lack of alexithymia [48], stress, social deficits [49], low self-esteem [50], resilience, mental health, mental and physical quality of life [51]. Studies have shown that alexithymia is positively correlated with loneliness in elderly patients with chronic diseases in the community, loneliness is also positively correlated with depression, and loneliness plays a partial mediating role between alexithymia and depression [52]. Some aspects of alexithymia may lead to mistrust, and this mistrust interacts with the initial alexithymia to produce a range of interpersonal problems that lead to loneliness [47]. The results of a path analysis study showed that narrative disorders can indirectly affect marital satisfaction through loneliness [53]. Numerous articles have started to examine the connection between these risk or protective factors and loneliness. According to several studies, loneliness, resilience, and mental health all have an impact on one’s physical and mental well-being [54]. Furthermore, higher degrees of loneliness are linked to worse levels of mental health and resilience. We discovered that resilience can lessen the negative effects of loneliness on a depressed person’s physical and mental health. According to some research, teenagers with poor emotional regulation experience more loneliness over time, whereas those with excellent emotional regulation are able to successfully combat loneliness by recognizing and controlling the negative emotions that go along with it [55]. Adolescence is a time of persistent loneliness, but as adolescents get older, they develop more mature interpersonal interactions, have more friends, and have fewer connections to their families. Teenagers’ experiences of loneliness change at this point, and this change will undoubtedly have an impact on other psychological variables that this study will examine.

### The current study

Much of the current research has focused on exploring risk factors or positive factors for the occurrence of NSSI in adolescents. Results showed that NSSI was associated with higher levels of alexithymia, depression, anxiety, bullying, impulsivity, substance abuse, history of abuse and sexual problems [56], as well as lower levels of mindfulness, resilience and self-esteem [26].

This study examines the potential mechanisms of loneliness, alexithymia, resilience and NSSI, focusing on whether psychological resilience can function as a protective mechanism against the onset of narrative disorders, loneliness and NSSI behaviors in adolescents. Can psychological resilience moderate feelings of loneliness among adolescents? By exploring these potential mechanisms, a theoretical basis can be constructed to develop an effective and widely applicable treatment plan for adolescents with depression and NSSI behaviors in the future.

## Methods

### Participants

This study is a nationwide multi-center combined cohort study of adolescent depression (CSCAD). Includes a cross-sectional study of adolescents with depression who are engaging in NSSI or have engaged in NSSI. A total of 2411 questionnaires were distributed, of which 2343 valid questionnaires were retrieved, with an effective recovery rate of 97.17%. The questionnaire was completed by 1826 girls and 517 boys, with an average age of (14.99 ± 1.65) years; the average number of years in education was (9.18 ± 1.76) years; 2135 participants were of Han ethnicity and 208 were from ethnic minority groups; about 67.4% of the participants lived in urban areas, while the remaining patients lived in rural areas; and the annual household income of the patients surveyed was (6.12 ± 2.44), expressed in units of 1,0000 yuan. Among the 2342 participants, 379 patients reported never having NSSI, and 182 patients had NSSI behaviors in the past but did not have NSSI in the last year, and 1782 patients had experienced NSSI in the past year.

### Procedure

From January to December 2021, adolescent patients with depression from 12 general hospitals and psychiatric hospitals across China were investigated and assessed using various clinical measurement instruments, and all participants provided their written informed consent. This paper has been approved by the Medical Ethics Committee of Shenzhen Kangning Hospital with the ethics number 2020-K021-02.

Inclusion criteria:

1. Participants met the diagnostic criteria of depressive episodes or depressive episodes corresponding to bipolar disorder as defined in the DSM-5; (outpatient depression status meets the criteria of depressive episode); 2) aged 12–18 years; 3) Years of education ≥ 6 years; and 4) the patients and their families agreed to participate in the research and signed the informed consent form.

Exclusion criteria:

(1) Patients with severe somatic diseases, infectious diseases, or immune system diseases; (2) patients with brain trauma, epilepsy, or other known serious neurological diseases or brain organic diseases; and (3) patients with a previous history of severe mental disorders such as schizophrenia or intellectual disability.

### Measures

#### General demographic data

Demographics information (age, sex assigned at birth, and ethnicity) was collected using a questionnaire created by the research team.

#### The functional assessment of self-mutilation (FASM) scale

The Functional Assessment of Self-Mutilation (FASM) scale is a structured interview questionnaire compiled by Lloyd in 1997. The Chinese version of the FASM scale was used in this study and showed good psychometric properties [57]. This paper mainly analyzes NSSI (e.g., cutting, burning, deliberately beating oneself) in order to understand whether the participants had intentionally exhibited 13 different behaviors associated with NSSI listed in the evaluation form over the past 12 months. Moreover, frequency, severity, treatment, and duration were assessed. In this study sample, Cronbach’s alpha was 0.991.

#### Toronto alexithymia Scale

The authors of the scale are Dr. Graeme J Taylor [58]. The translated scales were used in our study, which is divided into three dimensions: Difficulty identifying feelings (DIF); (2) Difficulty describing feelings (DDF); Externally-oriented thinking (EOT). With reference to a study on alexithymia in children and adolescents, it was noted that the EOT subscale has low reliability in adolescents. Loas et al. found that the scale at this point could provide a reliable and valid measure of emotional disturbance in adolescents when measured using the 12 items in the TAS-20 [59]. Therefore, data from the EOT subscale were excluded from the data processing of this study. Each item is scored according to a 5-point (1–5) scoring method. In this study sample, Cronbach’s alpha was 0.812.

#### UCLA loneliness scale

The first edition of this scale was compiled in 1978 by Russell et al. It was revised twice in 1980 and 1988 [60]. The current study used the third version of this scale [61], which has been shown to be effective in measuring subjects’ feelings of loneliness [52]. Each item is scored according to a 4-point (1–4) scoring method. It mainly evaluates feelings of loneliness that are caused by a gap between a desire for social communication and the actual level of communication. In this study sample, Cronbach’s alpha was 0.908.

#### Connor davidson resilience scale (CD-RISC)

The original version was compiled by Connor and consists of 25 entries [62]. This study used the Chinese version of the short-form of this scale compiled by Campbell [63]. It has been shown to be effective in measuring the resilience of subjects [38]. Each item is scored according to a 5-point (0–4) scoring method. In this study sample, Cronbach’s alpha was 0.921.

#### Patient health questionnaire-9 (PHQ-9)

The Depression Screening Scale (PHQ-9) is one of the internationally used depression screening scales. Its nine entries cover the DSM-5 diagnostic criteria for depressive disorders, making the scale both useful for assessing depression severity and potentially diagnostically valid [64–66]. In this study sample, Cronbach’s alpha was 0.904.

### Statistical analyses

SPSS version 21.0 (IBM Corp, Armonk, NY, USA) was used for statistical analysis. The counting data are expressed in [n (%)]. Firstly, for data validity, we examined the common method bias. Secondly, descriptive analyses were conducted for demographic information. Then, the Shapiro-Wilks test was used to test the normality of each continuous key variable. The total score of PHQ-9, CD-RISC-10, TAS and UCLA did not satisfy the normal distribution in our dataset. Therefore, the median (M) and inter-quartile range (IQR) were used to describe their distribution characteristics, and Spearman correlation analysis was carried out to evaluate the correlation between each variable. Finally, we tested the differences between the two groups using the Mann-Whitney non-parametric test.

Factors associated with NSSI in adolescents with depression were analyzed by binary logistic regression. The dependent variable is a dichotomous variable, 1 (engaging in NSSI), 0 (patients without NSSI and those who have had NSSI in the past but not in the last year). Independent variables included alexithymia, resilience, loneliness and depression. Mplus8 software was used for calculation, which was exploited by Linda Muthén and Bengt Muthén. We used the first stage regulation model of the seven moderated mediation models proposed by Edwards (2007) in the article published by psychological methods. NSSI was used as the dependent variable, alexithymia as the independent variable, resilience as the mediating variable and loneliness as the moderating variable put into the model for calculation. As the NSSI is a nominal variable, the type we have chosen is general, estimator is MLR, the confidence interval was 95%.

## Results

### Detection rate of NSSI

A list of the types and frequency of NSSI is shown in Table 1. In this survey, 2343 adolescent patients with depression were investigated. A total of 1782 cases of NSSI were reported in the past year, with a detection rate of 76.06%. Among them, the frequency of deliberate cutting or scratching of skin reached 87%, which was the most common method of self-harm. The second was deliberately punching, hitting, or slapping oneself (55.4%); followed by hitting the fist or head on a hard surface (50.2%), deliberately biting oneself (47.9%), and deliberately scratch one’s skin (44.4%).

**Table 1:** Types and frequency of NSSI

	rate n = 1782(%)	Frequency of occurrence
Deliberately cut or scratch your skin	1551(87.0)	77.75 ± 280.94
Deliberately hit yourself	988(55.4)	74.07 ± 174.36
Pull your hair on purpose	719(40.3)	88.24 ± 305.81
Deliberately use sharp objects to stab and engrave characters or patterns on the body	615(34.5)	41.22 ± 180.44
Deliberately stimulate the wound and hinder healing	684(38.4)	68.81 ± 206.56
Deliberately stabbing objects into the skin or nails	328(18.4)	69.91 ± 259.53
Deliberately bite oneself, such as the mouth or lips	853(47.9)	106.24 ± 377.24
Deliberately scratch yourself and bleed	562(31.5)	50.15 ± 199.02
Strike with your fist or head against a hard object	897(50.2)	66.52 ± 216.67
Deliberately scratch your skin	792(44.4)	83.39 ± 277.92

### Descriptive statistics and correlation

The correlation analysis of alexithymia, resilience, loneliness, and NSSI in adolescent patients with depression is shown in Table 2. Resilience was negatively correlated with depression, alexithymia, and loneliness (r = − 0.497–−0.577, P < 0.001). The total score of resilience was negatively correlated with NSSI (r = − 0.281, P < 0.001). Alexithymia and loneliness were positively correlated to NSSI (r = 0.223–0.276, P < 0.001).

**Table 2:** The correlation analysis between alexithymia, resilience, loneliness and NSSI

	M	IQR	Skewness	Kurtosis	1	2	3	4
1	1	0	1.486	0.422				
2	45	11	-0.722	0.625	0.247**			
3	13	11	0.705	0.375	-0.291**	-0.497**		
4	58	14	-0.707	0.375	0.223**	0.599**	-0.577**	
5	18	11	-0.559	-0.521	0.276**	0.604**	-0.549**	0.620**

Notes: M, mean; SD, standard deviation. *indicates a difference at the significance level of 0.05, **indicates a difference at the significance level of 0.01. 1.NSSI;2.alexithymia; 3.resilience; 4.loneliness; 5.depression.

The Mann-Whitney non-parametric test was performed to analyze whether the differences in alexithymia, loneliness, depression and resilience outcomes were significant between group 1 (patients who engaged in NSSI) and group 0 (patients who did not have NSSI and those who had had but not had NSSI in the past year) and the results showed statistical significance at p ≤ 0.05. The results revealed a significant difference in alexithymia between groups 1 and 0 (Z=-11.534, p < 0.001); that is, depressed adolescent patients with NSSI behaviors exhibited more severe narrative impairment. Loneliness was significantly different between groups 1 and 0 (Z=-10.536, p < 0.001), such that adolescents with NSSI behaviors had stronger feelings of loneliness. Resilience was significantly different between groups 1 and 0 (t=-13.861, p < 0.001); that is, depressed adolescents without NSSI behaviors exhibited greater psychological resilience. Depression was significantly different between groups 1 and 0 (t=-12.895, p < 0.001), such that patients with NSSI behaviors exhibited more severe levels of depression. The results are shown in Table 3.

**Table 3:** Differences in psychological factors between groups of adolescents with depression and without NSSI

	NSSI (M ± SD)		Z	P
	0 (n = 561)	1(n = 1782)		
alexithymia	40.42 ± 9.58	45.80 ± 8.38	-11.534	< 0.001
resilience	18.17 ± 8.80	12.39 ± 7.88	-13.861	< 0.001
loneliness	52.41 ± 12.07	58.37 ± 10.81	-10.536	< 0.001
depression	13.33 ± 7.42	17.97 ± 6.72	-12.895	< 0.001

Notes: M, mean; SD, standard deviation. NSSI is divided into two groups: 1: engaging in NSSI; 0: patients without NSSI and those who have had NSSI in the past but not in the last year

### NSSI related factors in adolescent depression

The results of the binary logistic regression are shown in Table 4. Alexithymia (B = 0.023, p = 0.003, OR = 1.023, 95% CI: 1.002 − 1.037) and depression (B = 0.045, p < 0.001, OR = 1.046, 95% CI: 1.026 − 1.066) when 1 (engaging in NSSI) and 0 (patients without NSSI and those who have had NSSI in the past but not in the last year) were compared, the implication is that there is a significant positive relationship between alexithymia and depression severity and NSSI, suggesting that depressed adolescents with higher scores on alexithymia and depression are more likely to engage in NSSI. Alexithymia and depression are risk factors for NSSI. Resilience (B = − 0.052, p < 0.001, OR = 0.949, 95% CI: 0.935 − 0.964), implying that higher levels of resilience have a significant negative relationship with NSSI, suggesting that adolescents with depression with higher resilience scores are less likely to engage in NSSI. Resilience is a protective factor for NSSI.

**Table 4:** NSSI related factors in adolescent depression

	B	S.E,	Wald*χ*^*2*^	P	*OR*	Lower bound (BC) 95% CI	Upper bound (BC) 95% CI
alexithymia	0.023	0.008	8.927	0.003	1.023	1.008	1.038
resilience	-0.052	0.008	47.274	< 0.001	0.949	0.935	0.964
loneliness	-0.006	0.006	0.770	0.380	0.994	0.982	1.007
depression	0.045	0.010	21.223	< 0.001	1.046	1.026	1.066

Notes: NSSI is divided into two groups: 1: engaging in NSSI; 0: patients without NSSI and those who have had NSSI in the past but not in the last year

### The mediated effect

**Fig. 1:**
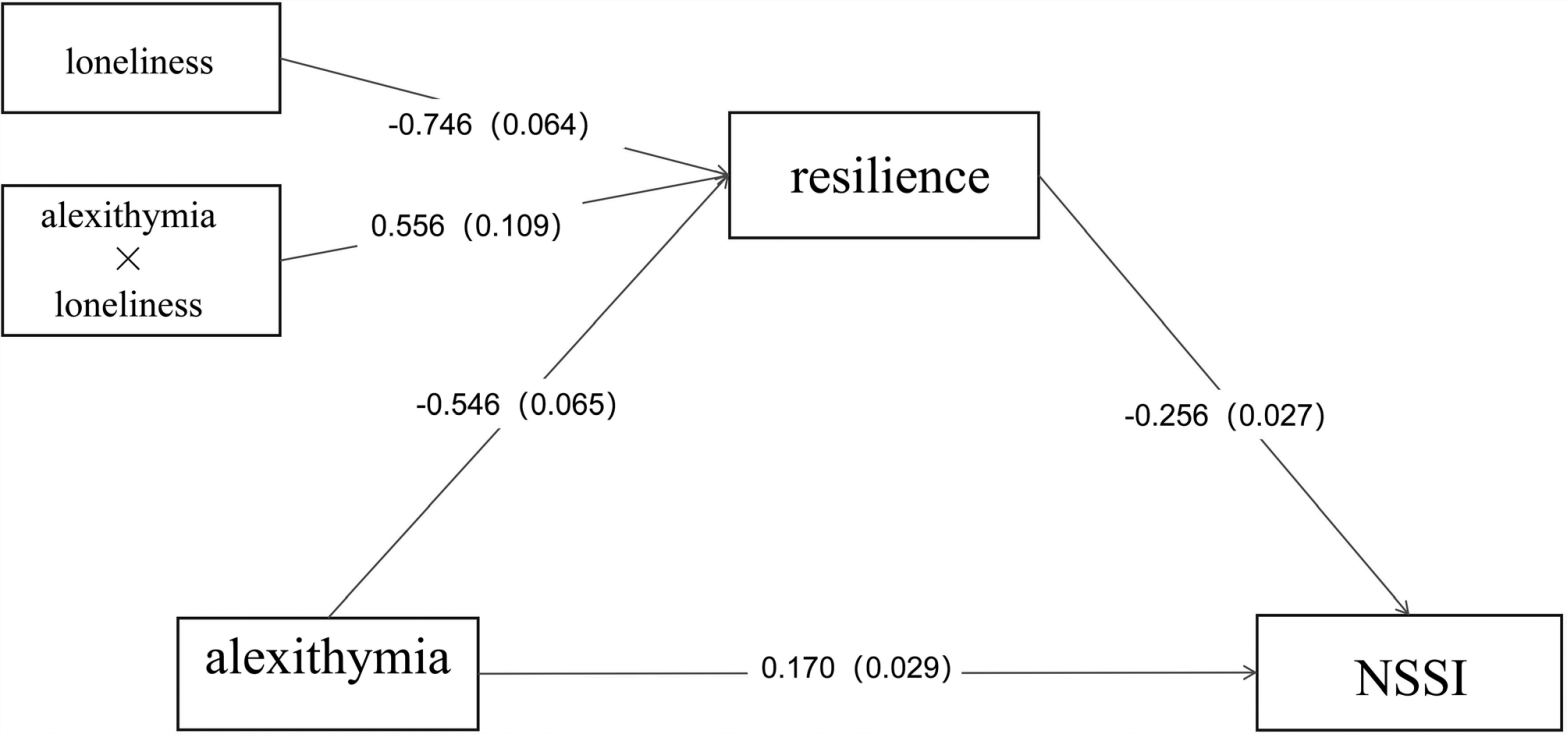
Mediating regulation model Note: The NSSI is divided into two groups. 1: engaging in NSSI; 0: patients without NSSI and those who have had NSSI in the past but not in the last year

A structural equation model was developed based on the results of the aforementioned correlation study, with alexithymia as the independent variable, resilience as the mediating variable, loneliness as the regulatory variable, and NSSI as the dependent variable. Table 5 displays the results. Alexithymia predicted NSSI positively (*β* = 0.170, t = 5.862, P < 0.001, 95%CI: 0.024 − 0.049), but negatively predicted resilience (*β*=−0.546, t = − 8.338, P < 0.001, 95%CI: −0.632−−0.393), l while loneliness predicted resilience negatively (*β*=−0.746, t = − 11.632, P < 0.001, 95%CI: −0.645−−0.460). The interaction term between alexithymia and loneliness was statistically significant (*β* = 0.556, t = 5.104, P < 0.001, 95%CI: 0.003 − 0.008), demonstrating that the moderating impact is still present. This moderated mediation model has a significant indirect effect (*β* = 0.030, t = 5.988, P < 0.001, 95%CI: 0.020 − 0.040). A simple slope analysis was performed to further understand the mediated effect of alexithymia and loneliness on resilience. Loneliness was divided into high and low subgroups by the mean plus or minus one standard deviation. The indirect effect was significant for the low loneliness subgroup (*β* = 0.031, t = 5.969, p < 0.001, 95%CI: 0.020 − 0.041), indicating that the indirect effect holds for the low subgroup and is significant for the high loneliness subgroup (*β* = 0.030, t = 6.007, p < 0.001, 95%CI: 0.020 − 0.040), The findings imply that, regardless of whether low or high values are used, the indirect influence of alexithymia on NSSI via resilience is substantial for loneliness as a moderating variable. Figure 1 depicts the outcome of this moderated mediation model.

**Table 5:** Moderated mediation models

				STDYX Standardization			t	p	Lower bound (BC) 95% CI	Upper bound (BC) 95% CI
				*β*		S.E.				
NSSI	alexithymia(c)		0.170		0.029		5.862	< 0.001	0.024	0.049
resilience	alexithymia(a1)		-0.546		0.065		-8.338	< 0.001	-0.632	-0.393
	loneliness(a2)		-0.746		0.064		-11.632	< 0.001	-0.645	-0.460
	alexithymia × loneliness(a3)		0.556		0.109		5.104	< 0.001	0.003	0.008
NSSI	resilience(b1)		-0.256		0.027		-9.436	< 0.001	-0.072	-0.046

Note: standard scores are used for all variables in this model; c is the direct effect of alexithymia - NSSI, a1 is the path coefficient of alexithymia - resilience, b1 is the path coefficient of alexithymia - NSSI, a2 is the path coefficient of loneliness - resilience, and a3 is path coefficient of loneliness × alexithymia - resilience. indirect effect: a1*b1. 95%CI:confidence intervals of model results

## Discussion

This study investigated the prevalence of NSSI among adolescents with depression, and further elucidated the relationship between loneliness, resilience, alexithymia and NSSI, with the ultimate goal of testing a moderated mediation model in which the relationship between alexithymia and NSSI is mediated by resilience, while loneliness moderates the relationship between narrative impairment and resilience.

The relationship between depression and NSSI is gaining traction [67]. Several studies have shown that depression is positively associated with NSSI [68], which is consistent with our findings (r = 0.276, p < 0.001). People who engage in NSSI are more likely to suffer from depression. Faced with the negative emotions and numbness associated with depression, sufferers usually self-harm [69].

The group reporting having NSSI had a considerably higher alexithymia score than those who had not reported experiencing NSSI in the year before. This suggests that, as compared to typical depressive patients, teenage depressed patients with NSSI have greater difficulties accurately identifying their emotions, demonstrate deficiencies in articulating their feelings, and have characteristics like rigid thinking and a lack of imagination. According to studies, people with severe mental illnesses are more likely to have NSSI [70]. The incidence of NSSI can be somewhat reduced in teenagers who have less alexithymia, according to research on emotion management-based group counseling [71]. Alexithymia scores were strongly and adversely linked with resilience scores, according to the findings (r = 0.577, p 0.001). As a result, lower resilience levels were linked to more severe alexithymia. The results imply that alexithymia can directly predict loneliness as well as many aspects of loneliness through interactions between those aspects and interpersonal distrust [47]. According to research, people with alexithymia may experience more loneliness as a result of deficiencies in emotional processing [72]. Individuals with alexithymia find it difficult to describe emotions, which results in indifferent interpersonal relationships and limited social networks, and thereby intensifies feelings of loneliness [73].

Resilience can produce protective mechanisms against NSSI and adversely predict it. The literature currently in print supports this conclusion [74]. Studies have shown that higher alexithymia and lower resilience are significant predictors of increased suicidal ideation during the first depressive episode [75]. This study discovered that the association between alexithymia and NSSI is mediated by resilience. NSSI is impacted by alexithymia both directly and indirectly through resilience. According to some research, empathy is a predictor of resilience in alexithymia, and residents’ resilience can be strengthened by improving their empathy, thereby decreasing alexithymia symptoms [76]. Loneliness also has a negative impact on resilience, according to research. The interplay of loneliness and alexithymia had a substantial influence on resilience as well. This suggests that loneliness has a strong moderating effect as a moderating variable in this moderated mediation model. Adolescents with alexithymia struggle to integrate into typical social situations and are more likely to feel lonely. And lonely people, due to a lack of vital social ties, will be unable to meet their emotional and belonging needs [77], exacerbating their alexithymia. According to some studies, the evolutionary hypothesis of loneliness argues that loneliness may work through a social distress mechanism that pushes people to repair and preserve social ties. Similarly, empirical research has indicated that lonely people are more likely to be driven to deal with problems involving social inclusion than non-lonely people and to enjoy better outcomes in such situations [78].

The Emotional Regulation Model emphasizes self-injury as a maladaptive coping strategy to reduce negative emotions and to free the individual from intolerable emotional states [79]. This is in line with the findings presented by Nock (2010), which showed that people minimize negative thoughts or feelings through self-injury, while negative thoughts or feelings also reinforce the use of self-injury, which is associated with negative emotions such as loneliness and depression [80]. Individuals with high levels of alexithymia primarily use primitive defenses, have limited empathic abilities, show deficits in mentalization and respond poorly to traditional interpretive psychotherapy [81]. This means that the treatment of depressed adolescents with NSSI and who exhibit high alexithymia needs to be further explored and improved in order to effectively alleviate NSSI behaviors and reduce the relapse rate. This mediated model with moderation suggests a possible therapeutic option. Resilience is defined as an individual’s ability to proactively adapt to their environment in the face of adversity, and it is comprised of two key conditions: exposure to significant adversity (e.g., exposure to community violence, parental mental illness, and poverty) and positive adaptation (good academic performance, positive relationships with teachers or peers)[82]. The Protective Resilience Model proposes that social support and loneliness can be effectively mediated through the agency of personal resilience, enabling individuals to have a better quality of life [83]. Considering the limited resources of psychiatrists and psychotherapists, parents and teachers can, in their daily lives, enhance the psychological resilience of adolescents and thus the psychological intervention aspect of adolescent depression with NSSI. It is also possible to follow the psychological developmental characteristics of adolescents who seek independence, and thus loneliness. In this sense, it is important to organize group activities in an effort to teach empathy, emotional expression and other skills, so as to alleviate feelings of loneliness, promote a sense of belonging and well-being, and equip such adolescents with alternative methods of emotion regulation, thus reducing the occurrence of NSSI.

## Conclusion

The advantage of this paper is that a large amount of sample data was collected from many areas across the country. There are many factors that affect NSSI; however, this article is the first to link alexithymia, loneliness, and resilience, and explore the underlying mechanisms of this association. By examining this potential mechanism, we hope to provide psychotherapists with a new way of thinking when carrying out treatment interventions with this population. At this stage, the treatment methods for NSSI among Chinese adolescents are very limited, and many children are unwilling and afraid to go to the hospital. If school teachers can understand the relationship between these influencing factors and learn relevant theoretical knowledge, they can also undertake intervention measures.

The main limitation of this study is that cross-sectional data were collected and a correlation analysis rather than a causal analysis was performed. In addition, the sample population was quite narrow in scope, and there are many potential NSSI patients that need our attention. In the future, it is hoped that some scholars can apply positive psychology as a psychological intervention to conduct longitudinal comparisons of patients, and analyze the causal relationship between potential mechanisms. In this way, research can focus on developing an intervention method that is applicable to clinical settings, daily learning, and daily life in order to help these patients, while encouraging teenagers solve problems and improve their mental health.

## Data Availability

The data that support the findings of this study are available from Hefei Fourth People’ Hospital but restrictions apply to the availability of these data, which were used under license for the current study, and so are not publicly available. Data are, however, available from the authors upon reasonable request and with permission of Shenzhen Kangning Hospital. To obtain the data in this study, the researchers may be contacted at yangxi12202022@126. com.
